# Muscle inversion closure technique with novel clips minimizes submucosal dead space after colorectal endoscopic submucosal dissection

**DOI:** 10.1055/a-2612-3542

**Published:** 2025-07-04

**Authors:** Taisuke Inada, Yorinobu Sumida, Tatsuya Matsumoto, Shin-ichiro Fukuda, Kosuke Maehara, Hirotada Akiho, Eikichi Ihara

**Affiliations:** 1 37060 Department of Gastroenterology, Kitakyushu Municipal Medical Center, Kitakyushu, Fukuoka, Japan; 2 12923 Department of Medicine and Bioregulatory Science, Graduate School of Medical Sciences, Kyushu University, Fukuoka, Japan

Endoscopic submucosal dissection (ESD) of the colorectum is technically demanding, with postoperative complications such as delayed perforation and bleeding necessitating reliable closure of the resection site 1 . Although various closure methods have been developed 2 , many focus solely on approximating the mucosal layer, often resulting in submucosal dead space (SDS), which may increase the risk of adverse events.

We previously reported a novel gastric closure method using a specialized clip with sharp claws and a strong gripping force to directly approximate the muscle layer, effectively reducing SDS 3 ( Fig. 1 ). In the present case, we applied this approach to the colorectum ( Video 1 ).

**Fig. 1:**
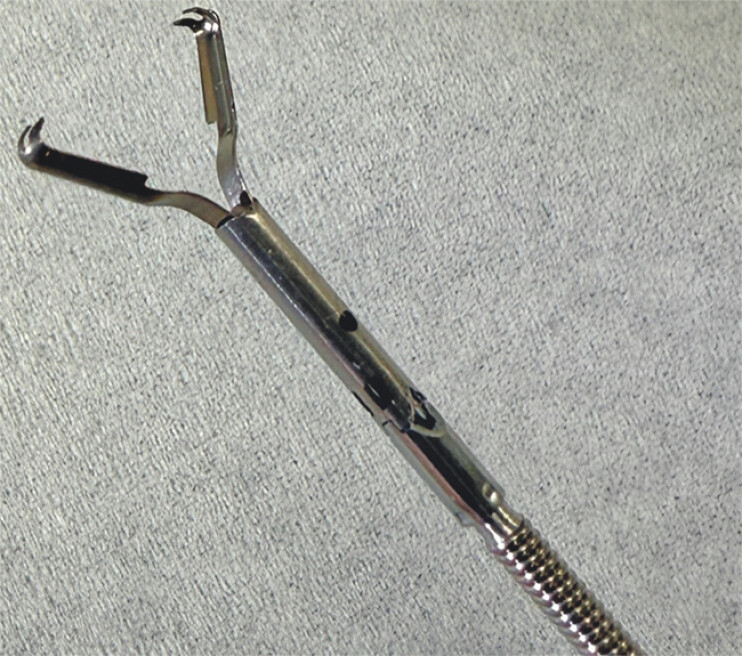
A new clip with sharp claws and strong gripping force to close the muscle layer.

Muscle layer closure with novel clips minimizes SDS after colorectal ESD.Video 1

A 72-year-old man underwent ESD of a 35-mm rectosigmoid neoplasm ( Fig. 2 **a** ). The muscle layer at one edge of the wound was first hooked to the claw of the clip underwater and guided to the opposite muscle layer ( Fig. 2 **b** ). After confirming alignment, strong suction was used to pull the tissue into the hood, followed by clip deployment ( Fig. 2 **c** ). This maneuver inverted and securely approximated the muscle layers, thereby eliminating SDS ( Fig. 2 **d** ). Additional conventional clips were used to close residual gaps ( Fig. 2 **e, f** ). The procedure was completed in 19 min without complications, and the patient was discharged uneventfully.

**Fig. 2:**
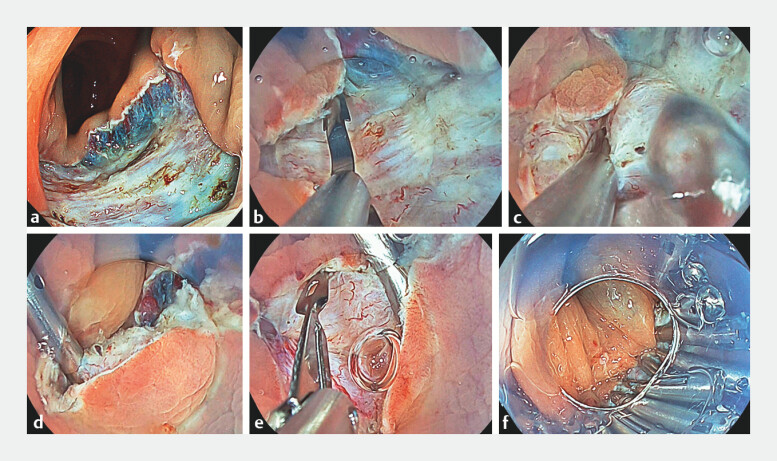
**a** The rectal colon tumor was resected without intraoperative incidents. **b, c, d** The clip’s sharp claws firmly engaged the muscle layers on both edges of the wound, effectively securing the tissue in a single deployment. By repeating the same technique, the wound was closed with minimal submucosal dead space. **e, f** Additional regular clips were placed to close gaps between the previously positioned clips, ensuring complete fixation of the muscle layer.

Several reports have described double-layered closure techniques in which both the muscle and mucosal layers are approximated 4 5 . These techniques share conceptual similarities with our proposed approach. We acknowledge that our method was partially inspired by these prior techniques. However, unlike double-layered methods, our approach prioritizes the secure closure of the muscle layer alone, minimizing the need for additional mucosal closure. We are currently conducting a prospective observational study to assess the safety and efficacy of this technique.

Endoscopy_UCTN_Code_TTT_1AQ_2AK

## Notes

Endoscopy E-Videos https://eref.thieme.de/e-videos
*E-Videos* is an open access online section of the journal *Endoscopy* , reporting on interesting cases and new techniques in gastroenterological endoscopy. All papers include a high-quality video and are published with a Creative Commons CC-BY license. Endoscopy E-Videos qualify for HINARI discounts and waivers and eligibility is automatically checked during the submission process. We grant 100% waivers to articles whose corresponding authors are based in Group A countries and 50% waivers to those who are based in Group B countries as classified by Research4Life (see: https://www.research4life.org/access/eligibility/ ). This section has its own submission website at https://mc.manuscriptcentral.com/e-videos .
